# GZFLW Induces Apoptosis of Ectopic Endometrial Stromal Cells via Promoting VPS53 Protein Stability

**DOI:** 10.1155/2018/1293630

**Published:** 2018-12-13

**Authors:** Ziyu Zhang, Faying Liu, YunYun Xu, Huang Huang, Yang Zou, Bicheng Yang, Yong Luo, Quan Zhang, Anwen Xiong, Liqun Wang, Ouping Huang

**Affiliations:** ^1^Key Laboratory of Women's Reproductive Health of Jiangxi, Jiangxi Maternal & Child Health Hospital, Nanchang, Jiangxi, 330006, China; ^2^Graduate School of Nanchang University, Nanchang, Jiangxi 330031, China; ^3^Department of Cardiac Surgery, The First Affiliated Hospital, Nanchang University, Nanchang, Jiangxi, 330006, China; ^4^Department of Medical Oncology, Shanghai Pulmonary Hospital & Thoracic Cancer Institute, Tongji University School of Medicine, Shanghai, 200433, China; ^5^Division of Obstetrics, Jiangxi Maternal & Child Health Hospital, Nanchang, Jiangxi, 330006, China

## Abstract

Endometriosis is still a major problem in obstetrics and gynecology. While GZFLW (Gui Zhi Fu Ling Wan) has been originally used for treating gynecological diseases, however, the molecular mechanism that GZFLW acts on endometriosis is not clear. To investigate the molecular mechanism that GZFLW plays role on endometriosis, iTRAQ (isobaric tags for relative and absolute quantification) proteomics and human endometrial stromal cells (Y14) obtained from a patient with endometriosis were used in in vitro study. Our results demonstrated that GZFLW decreased Y14 cells proliferation while increased cells apoptosis. The differential expression protein VPS53 (Vacuolar protein sorting 53 homolog) was predicted by iTRAQ coupled LC-MS/MS and further identified by western blot. Besides, GZFLW induced VPS53 protein level by promoting its stabilization. Our findings highlight a novel role for VPS53 in gynecology and provide a potent therapeutic strategy against endometriosis.

## 1. Introduction

Endometriosis is the presence of endometrial cells in other parts outside the uterine cavity, such as ovaries, fallopian tubes, bladder, and myometrium [1]. The main pathological changes are ectopic endometrial bleeding and the surrounding tissue fibrosis. The main symptoms of endometriosis are the formation of ectopic nodules, dysmenorrhea, chronic pelvic pain, abnormal menstruation, and infertility, which are seriously affecting the health and quality of life of young women [2–6]. Because its pathogenesis is unknown, it is still a major problem in obstetrics and gynecology.

There is a long history of traditional Chinese medicine, which has contributed to the prevention and treatment of various diseases. Gui Zhi Fu Ling Wan (GZFLW) is one of the formulations in the ancient Chinese medicine and is composed of five crude drugs, including Cinnamomi Cortex, Poria cocos, Moutan Cortex, Persicae Semen, and Paeoniae Radix. While GZFLW has been originally used for the treatment of gynecological diseases [7], however, the molecular mechanism that GZFLW acts on endometriosis is not clear.

Here, we isolated and cultured the primary ectopic endometrial stromal cells (EESCs, Y14) [8], adding GZFLW-containing serum for processing, then used the iTRAQ proteomics approach, and found the differential expression protein VPS53. Our results demonstrate that GZFLW can increase the expression of VPS53 through promoting its stability and induce the apoptosis of endometriosis stromal cells via mitochondria-induced apoptotic pathway.

## 2. Materials and Methods

### 2.1. Isolation of Human Ectopic Endometrial Stromal Cells

Ectopic endometrial tissue from a patient without hormone treatment for more than 3 months undergoing hysterectomy from the Jiangxi Maternal and Child Health Hospital (Nanchang City, Jiangxi Province, China). The sample has been shown to be in the proliferative phase of the menstrual cycle in pathology and histology. The patient signed a written informed consent form prior to recruitment. This study is in line with the Helsinki Declaration and approved by the Ethics Review Body Committee of the Jiangxi Maternal and Child Health Hospital.

Separation, characterization, and culture of EESCs were performed as described previously [8–10]. In short, the tissue were cut into small pieces and incubated with 10% FBS and DMEM/F12 supplemented with 1 mg/ml type IV collagenase for 1.5 hours at 37°C. The tissue was digested and filtrated with a cell strainer. The cell was centrifuged at 1000 rpm for 5 minutes. The pellet was resuspended and cultured with DMEM/F12 containing 100 IU/ml penicillin, 50 mg/mL streptomycin, and 10% FBS. In our previous work [8], cell characterization was assessed by IHC using anti-Vimentin (1:70 dilution, Zhongshan Jinqiao Biotec, China) and anti-Cytokine (1:100 dilution, Zhongshan Jinqiao Biotec, China) antibodies. In the present work, the purity EESCs >95% and cells were used between passages 3 to 10. We named the purity cell Y14.

### 2.2. Preparation of Serum Containing GZFLW

After the approval by the Ethics Committee of Jiangxi Maternal and Child Health Hospital (Nanchang, Jiangxi Province, China), the Experimental Animal Center of Nanchang University provided female Sprague Dawley rats weighing 180-200g. After removal of the capsules, the GZFLW powder was dissolved in sterile 0.5% sodium carboxymethylcellulose. The study was conducted in accordance with the guidelines from Nanchang University for the care and use of laboratory animals. Each group contained 10 rats which were used to obtain serum with or without GZFLW. Rats in the two groups were treated with normal saline or 1.2 g / kg GZFLW for 7 days, respectively. Blood was obtained aseptically from the abdominal aorta of the rat 2 hours after the final administration, and then the blood was centrifuged at 2500 rpm for 20 minutes to obtain serum. After filtering twice with a 0.22 *μ*m cellulose acetate membrane, the serum was bottled, precipitated in water at 56°C for 30 minutes, and stored at -20°C for use [11].

### 2.3. Cell Viability Detection

The cell viability was measured by the CCK-8 kit (Bestbio, China Co., Ltd.) and determined for 4 days. The cells after 72 hours of infection were seeded in a 96-well plate at about 6.0x10^3^ cells/well. 10*μ*l of CCK-8 solution was added to the medium, and the Y14 cell was incubated at 37°C for 2 hours. The proliferation rate of Y14 cell was detected at 0, 24, 48, 72, and 96 after infection. Optical density (OD) value was measured at 450 nm (iMarkTM Microplate Reader, Bio-Rad, USA). All of these experiments were repeated three times.

### 2.4. Western Blot and Protein Turnover Assay

Western blotting was performed as previously described [12, 13]. To measure the turnover rate of endogenous VPS53 protein level, Y14 cells were treated with or without GZFLW- containing serum, respectively. After 48 hours, the cells were treated with cycloheximide (CHX, 20 um, Sigma, protein synthesis inhibitor). At each time point, RIPA buffer (50 mM Tris-HCl, pH 7.5, 150 mM 475 M NaCl, 1% Nonidet P-40, 0.5% sodium deoxycholate, 0.1% SDS, 1 x Roche complete protease inhibitor mixture) was used to lysis cell. Protein (30 *μ*g/line) was separated by SDS-PAGE gel and transferred onto a PVDF membrane (Millipore, CA, USA), then blocked with 5% milk powder, and incubated with anti-VPS53 antibody (1:1000 dilution, rabbit, ab106469, Abcam) overnight at 4°C. The membrane was washed three times with 1xTBS-T and incubated with horseradish peroxidase (1:5,000) secondary antibody for 1 hour at room temperature. Protein bands were visualized by ECL system (Thermo Fisher Scientific, IL, USA). VPS53 expression levels were normalized to the expression of *β*-actin in each line.

### 2.5. RNA Isolation and qPCR

We carried out the RNA isolation as described previously [13, 14]. The total RNAs were extracted from Y14 cells by using the RNAiso reagent (TaKaRa, Shiga, Japan). The reverse transcription reaction was also performed by using the PrimeScript reagent Kit (TaKaRa). Real-time quantification PCR reaction was carried out by using the SYBR Premix Ex TaqII (TaKaRa) on the 7500 Real-Time PCR System (Applied Biosystems) with primers for RT-hVPS53-F′: CCGAGAATACGCCTGGAAAA, RT- hVPS53-R′: ATGTTACAGATGAGGCAGAGC. RT-hActin-F′: ACCTTCTACAATGAGCTGCG, and RT-hActin-R′: CCTGGATAGCAACGTACATGG. Experiments were repeated at least three times.

### 2.6. Flow Cytometric Analysis with Annexin V/PI Assay System

Flow cytometric analysis was performed as described previously [15]. Cold 1xPBS was used to wash Y14 cells. The Y14 cell was resuspended in the staining buffer and detected with Annexin V-FITC Apoptosis Detection kit (Bestbio Co., China) according to the manufacturer's instructions. FACS Cytomics FC 500 MCL flow cytometer was used to analyze the stained cells (Beckman Coulter, USA).

### 2.7. EdU Incorporation Assay

After with or without GZFLW treatment, Y14 cells were seeded onto 24-well plate. Next day, cells were incubated with 10 *μ*M Edu for 6 hours, washed 3 times wtih 1xPBS, fixed in 3.7% formaldehyde for 15 min at room temperature, and followed by Edu insertion detection according to the manufacturer instructions (Guangzhou RiboBio Co., Ltd, China) [13].

### 2.8. The Lentiviral Constructs

The lentiviral were purchased from Guangzhou Cyagen Biosciences Co., Ltd, Guangzhou, China. The three shVPS53 sequences are 1#CACTCAGTTCTGCGTTAAATT; 2#GAAAGAAATCACCCGTGATAT; 3#CCAGAAGTACCTCCGAGAATA. The oligo was inserted into pLV[shRNA]-Puro-U6 vector.

### 2.9. iTRAQ-MS/MS Analysis

The iTRAQ-MS/MS was carried out at the Shanghai Applied Protein Technology Co., Ltd, China [16]. Protein was digested according to the FASP procedure described by Wisniewski [17]. The 8-plex iTRAQ reagent was used to label the resulting peptide mixture as described by the manufacturer's instructions (Applied Biosystems).

### 2.10. Statistical Analyses

Statistical analyses were carried out by using SPSS 24.0 software. Data were presented as mean ±SEM. To determine the statistical difference of VPS53 mRNA expression between shControl group and shVPS53 group by using Student's t-test, all p-values were two-tailed, and p-values < 0.05 were considered to statistical significance (*∗*).

## 3. Results

### 3.1. GZFLW Decreased Y14 Cells Proliferation While Increased Cells Apoptosis

**Figure 1:**
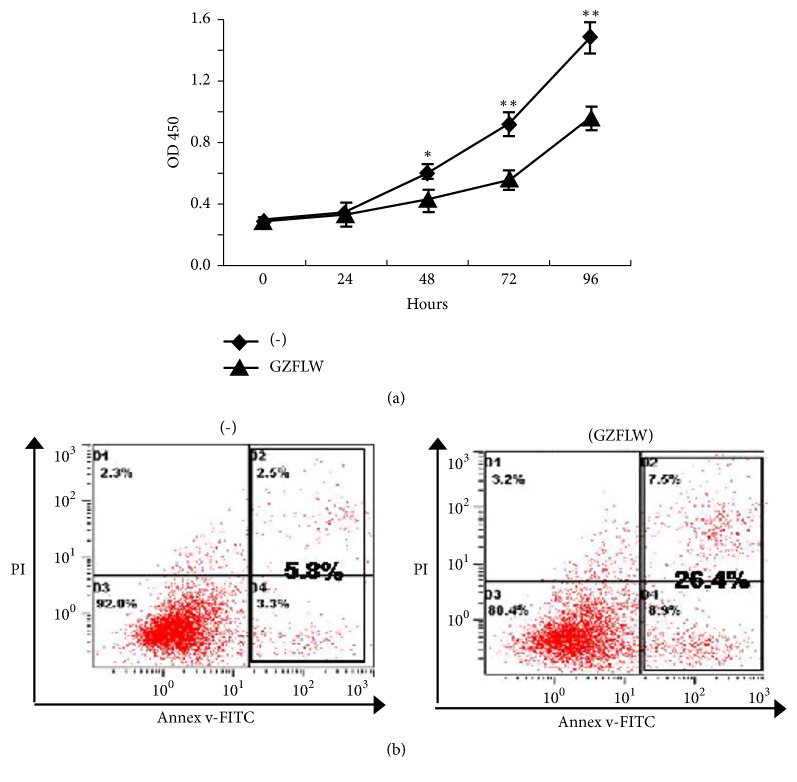
GZFLW promotes endometrial stromal cell apoptosis. (a) Endometrial stromal cells were treated with blank serum and GZFLW-containing serum, and the viability of the cells was measured continuously for 5 days using the CCK-8 method. Values are means ± SEM.* p*-values were determined by Student's t-test, *∗p* < 0.05 and *∗∗p* < 0.01. (b) The effect of GZFLW on apoptosis was stained with Annexin V-FITC and prodiduim iodine (PI) and detected by FACS.

**Figure 2:**
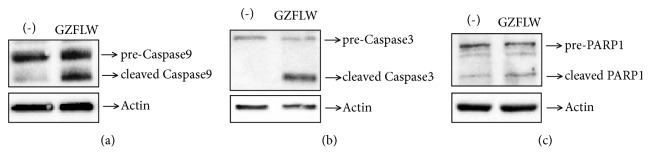
GZFLW induced apoptosis via mitochondria-initiated pathway. (a-c) Y14 cells were treated with GZFLW for 48 hours; the cleavages forms of Caspase-9 (a), Caspase-3 (b), and PARP1 (c) were detected by western blot. Actin as an internal control.

We detected the Y14 cells viability by Cell Counting Kit-8(CCK-8) assay after GZFLW treatment for 0, 24, 48, 72, and 96 hours. The value of OD450 in control group was higher than the group treated by GZFLW after 48 hours (Figure 1(a)), indicating that GZFLW could decrease the Y14 cells viability. Apoptosis is one of the factors that decrease cell viability. To determine the possible contribution of apoptosis to GZFLW-mediated cell growth inhibition, Y14 cells were treated with GZFLW for 48 hours and assessed by Annexin V-FITC/prodiduim iodine (PI) double staining. As shown in Figure 1(b), GZFLW treated cells exhibited a significant increase with Annexin V staining (16.4% in GZFLW group compared with 5.8% in untreated cells). To evaluate the role of caspase in GZFLW-induced apoptosis, cleavages of caspase-3 and caspase-9 were detected by western blot. As shown in Figures 2(a) and 2(b), both caspase-3 and caspase-9 were processed after GZFLW treatment for 48 hours. We then tested the cleavage of the poly(ADP-ribose) polymerase (PARP), which is a caspase substrate. As expected, its cleaved band was observed after GZFLW treatment (Figure 2(c)).

### 3.2. GZFLW Induced VPS53 Protein Level by Promoting Its Stabilization

**Figure 3:**
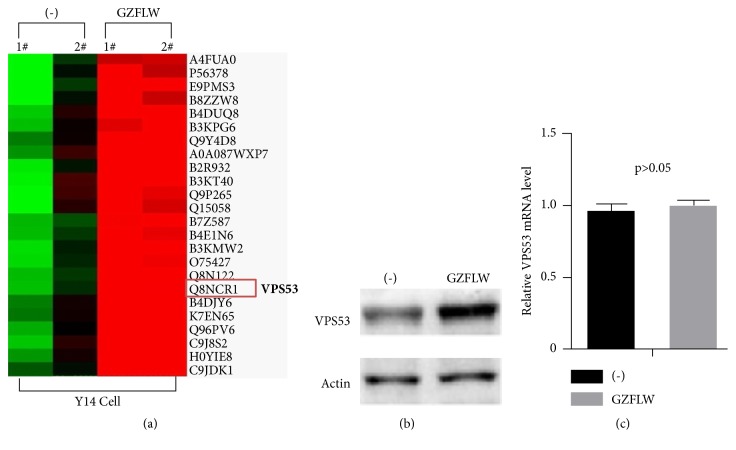
GZFLW induces VPS53 protein expression level in Y14 cells. (a) Part of the differential expression proteins by the iTRAQ proteomics, including the VPS53 protein. (b) Total protein was extracted from Y14 cells with or without GZFLW treatment; western blot was performed using anti-VPS53 antibody, and Actin was used as an internal control. (c) Total RNA was extracted after treatment of Y14 cells with or without GZFLW, and qRCR was performed to detect the mRNA expression of VPS53. *p* > 0.05 means that there was no statistical significance.

**Figure 4:**
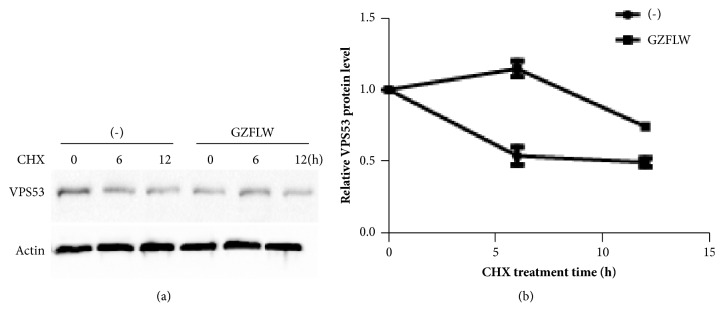
GZFLW promotes stability of VPS53 protein. (a) Western analysis of VPS53 in Y14 cells following cycloheximide (CHX, 20um) treatment in the presence of GZFLW or not. Actin as an internal control. (b) Time course of VPS53 turnover experiments as described in (c). The intensity of VPS53 bands relative to that of Actin from each time point was quantified by ImageJ software package and plotted against the incubation time.

**Figure 5:**
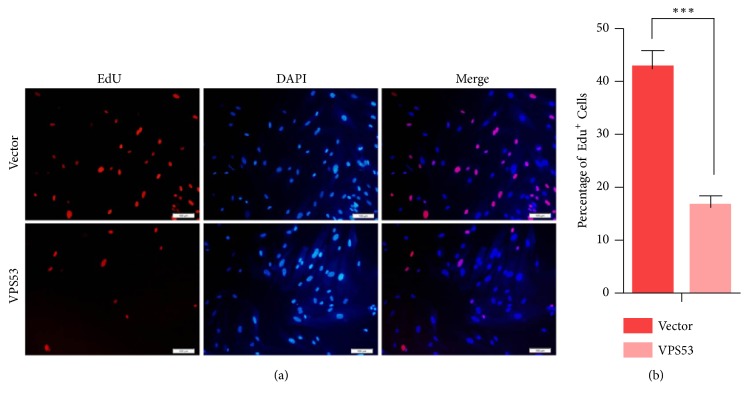
Overexpression of VPS53 decreases Y14 cells proliferation. The percentage of EdU positive cells was blindly calculated with counting six nonoverlapping fields. Scale bar=100*μ*m. Values are means ± SEM. P-values were determined by Student's t-test, *∗∗∗p* < 0.001.

To investigate the molecular mechanism of how GZFLW influences the apoptosis, we applied iTRAQ (Isobaric Tags for Relative and Absolute Quantitation) [18–20], a high-throughput strategy to find out the differential expression level of target proteins regulated with or without GZFLW treatment. iTRAQ analysis of GZFLW treated Y14 cells resulted in identification of 5512 unique proteins, of which 97 were downregulated (Fold Change<0.8, p<0.05) and 275 upregulated (Fold Change>1.2, p<0.05) as compared with negative control. Among them, VPS53 (also named HCCS-1), a Golgi-associated retrograde complex (GARP) subunit, had significantly increased with GZFLW treatment (1.7 fold change) compared with untreated Y14 cells (Table 1 and Figure 3(a)). Previous work suggested that overexpression of VPS53 induced apoptosis via mitochondria-initiated pathway [21, 22] and overexpression of VPS53 by infecting lentivirus into Y14 cells to perform EdU insertion experiments; we found VPS53 overexpression decreased Y14 cells proliferation (Figure 5). Therefore, we hypothesized that GZFLW induced Y14 cells apoptosis through VPS53 mediated mitochondria-initiated pathway. To prove our idea and validate the iTRAQ Proteomics result, we measured the protein level of VPS53 with or without GZFLW treatment. As shown in Figure 3(b), VPS53 protein level was upregulated by GZFLW. However, the mRNA level of VPS53 was not significant changed by GZFLW treatment (Figure 3(c)). This data indicated that GZFLW induced VPS53 expression level through promoting VPS53 post transcriptional level. Besides, the VPS53 protein level was not altered after GZFLW treatment in normal endometrial cell (Supplementary material (available here)). Ubiquitin-mediated proteasome degradation decreases protein posttranscriptional level and stability. Therefore, we utilized cycloheximide (CHX, 20um) to block protein synthesis. As shown in Figures 4(a) and 4(b), under the condition of without GZFLW treatment, VPS53 protein level rapidly reduced to half of its original amount at 6 h, but no significant change was observed at this time point with GZFLW treatment. These results suggested that GZFLWinduced VPS53 protein level by promoting its stabilization.

### 3.3. GZFLW Promoted Y14 Cells Apoptosis through VPS53 Protein

**Figure 6:**
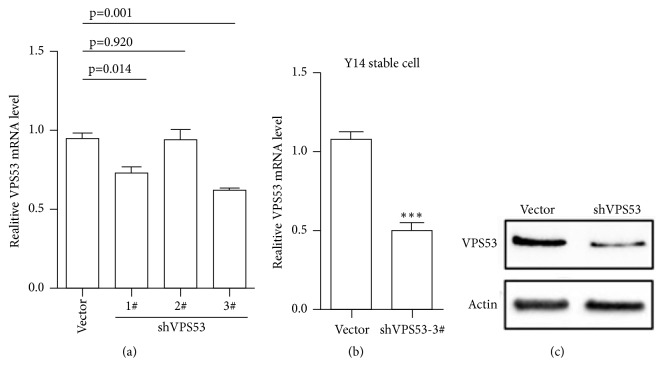
Using shRNA to konck down VPS53 and validated by RT-PCR and western blot. (a) Control virus vector, shRNA-1 #, 2 #, and 3 # lentiviruses are transiently infected in Y14 cells and total RNA is collected 72 hours later for qPCR detection. (b) Screening of stable cell lines using the third shRNA (3 #), and the mRNA expression of VPS53 was detected by qPCR. *∗p* < 0.05.

**Figure 7:**
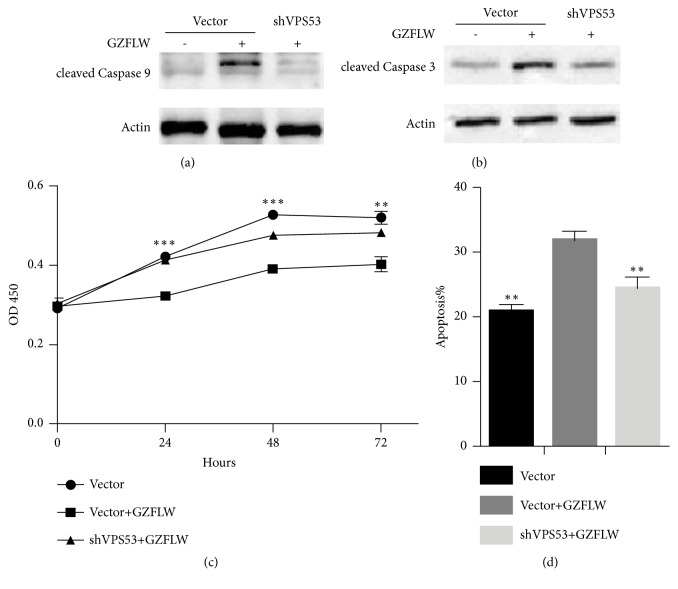
GZFLW activates Caspase-3 and Caspase-9 via VPS53. (a and b) Western blot detection of activated Caspase-9 and Caspase-3 expression in shControl stable Y14 cell lines and shVPS53 stable cell lines with or without GZFLW treatment, Actin as an internal control. (c) CCK-8 assay detected the Y14 cells proliferation of three groups at 24, 48, and 72 hours, respectively. (d) Cells in three groups were stained with Annexin V-FITC and prodiduim iodine (PI) and detected by FACS. Values are means±SEM.* p*-values were determined by Student's t-test, *∗∗p* < 0.01 and *∗∗∗p* < 0.001.

To investigate if GZFLW mediates apoptosis of Y14 cells via VPS53, we need to construct a stable cell line that knockdown VPS53. We ordered three VPS53 lentiviral knockdown vectors (# 1, # 2, and # 3, respectively) that were infected into Y14 cells. The level of mRNA of VPS53 was examined and the third shRNA was found to be most effective (Figure 6(a)). After that we used puromycin (2ug/ml) for screening stable cell line and finally yielded a Y14 cell line that stably knocked down VPS53 (Figure 6(b)). We then added GZFLW to control stable Y14 cells for 24 hours. After total protein was extracted, the cleavages forms of Caspase-3 and Caspase-9 were detected by western blot and found to be increased (Figures 7(a) and 7(b)). Subsequently, GZFLW was added to Y14 cells stably knocking down VPS53, and both activated forms of Caspase-3 and Caspase-9 were found to be reduced (Figures 7(a) and 7(b)). The above results indicate that GZFLW activates Caspase-3 and Caspase-9 through VPS53.

Finally, to further confirm the effect of GZFLW on the regulation of cellular function by VPS53, we used CCK-8 to detect cell viability and PI-Annexin V-labeled flow cytometry to determine apoptosis. It was found that when GZFLW was added to Y14 cells stably knocking down VPS53, both cell proliferation and apoptosis recovered to the control level comparing with GZFLW treatment alone (Figures 7(c) and 7(d)).

## 4. Discussion

GZFLW, as a traditional Chinese medicine, owns many years of history for the treatment of endometriosis and has also been used for other diseases in animal models, such as dietary obesity in rats [23], improvement of microcirculation in mice [24], and inhibition of platelet aggregation in Guinea Pig [25]. However, due to the complexity of traditional Chinese medicine ingredients, its molecular biology mechanism is not yet clear. Here, we added GZFLW-containing serum into endometriosis stromal cells (Y14) and used proteomic techniques (iTRAQ) and mass spectrometry (LS/MS) to find the key differential expression protein in order to uncover its mysterious veil.

Firstly, we added GZFLW's drug-containing serum to endometriotic stromal cells (Y14) and found that it could inhibit cell growth while promoted cell apoptosis. The mitochondrial pathway is one of the main pathways of apoptosis. The activation of Caspase-9 initiates a cell death pathway, which in turn amplifies and transmits the apoptotic signaling cascade leading to the activation of Caspase-3 and the promotion of PARP cleavage, resulting in DNA damage, cell instability, and apoptosis process [26, 27].

Through iTRAQ, we found a key protein VPS53 (vacuolar protein sorting 53 homolog), which is a subunit of the Golgi-associated retrograde protein (GARP) complex. The reasons we considered VPS53 as a key protein are as follows: (1) GZFLW can promote the expression of VPS53 and is 1.7 times higher than that of the control group, and it has statistical significance; (2) It has been reported that VPS53 can induce the release of cytochrome by inserting BAX into the mitochondrial membrane, which triggers the mitochondrial pathway and promotes cell apoptosis [21, 22]; (3) Inducing apoptosis of endometriosis stromal cells could suppress endometriosis progression [28–32]. Based on this, we speculate that GZFLW may promote the apoptosis of cells by upregulating the expression of VPS53, thereby inhibiting the growth of endometriotic stromal cells. Then we used western blot to determine the differential expression protein VPS53 found in iTRAQ experiments and it was indeed upregulated after treated with GZFLW-containing drug-containing serum, but its mRNA did not change. Therefore, we concluded that GZFLW promotes VPS53 expression at posttranscriptional translation level. By using CHX (a protein synthesis inhibitor) to detect the turnover rate of VPS53 protein, we found that the half-life of GZFLW treated group was higher than that of the control group, indicating that GZFLW can improve the stability of VPS53. It is unclear how GZFLW improves the stability of VPS53. It has been reported that VPS52, one of the members of the GARP complex, binds to the E3 ubiquitin ligase RNF41 and promotes the degradation of VPS52 by the proteasome [33]. GZFLW may play a role in promoting the stability of VPS53 by inhibiting the enzyme activity of RNF41 or other E3 ubiquitin ligase. Whether GZFLW indeed promotes apoptosis through VPS53 needs to be confirmed by loss of function experiment. We added GZFLW to stable Y14 cells that knocked down VPS53 and found that GZFLW lost its function to induce apoptosis, indicating that VPS53 is essential for GZFLW promoting apoptosis.

Tu Youyou won the Nobel Prize for the treatment of malaria due to the extraction of artemisinin from Chinese herbs. At present, the role of Chinese herbal compound has been recognized in East Asia and even Europe and the United States. However, there is little knowledge about the mechanism of Chinese herbal compound. Therefore, it is very important to study the molecular mechanism of the Chinese herbal compound. The serum pharmacology is one of the methods for the study of traditional Chinese medicine compound prescriptions [34]. It can reflect the pharmacological effects of traditional Chinese medicines in vivo and is suitable for in-depth study of traditional Chinese medicine compounds at the cellular level. In this study, Y14 cells were treated with GZFLW-containing serum to avoid the interference of physicochemical properties of GZFLW compound crude preparations in vitro and to reflect the metabolites produced by the drug components. The differential expression protein VPS53 discovered in our study through iTRAQ technology can be used as a target for the treatment of endometriosis in the future.

## Figures and Tables

**Table 1:** Ten significant, replicating upregulated proteins identified in iTRAQ. As a selection criterion, protein confidence p<0.05 (analyzed by Protein discoverer software 1.4) was used. The table includes protein accession number, protein name, ratios, number of peptides used for quantitation, and p values of expression between Control and GZFLW group, average protein expression (fold change Control vs. GZFLW).

**Accession**	**Description**	**Peptides**	**GZFLW-1#/ Control -1#**	**GZFLW-2#/ Control -2#**	**Average **GZFLW/** Control**	***p* values**
**I3L0Y5**	ATP synthase	1	3.04	2.87	**2.95**	6.25E-53
**C9JF90**	UPF0415 protein	1	2.89	2.44	**2.67**	9.64E-44
**Q9P0C7**	HSPC254	1	2.63	1.99	**2.31**	2.12E-32
**O00172**	Line-1 reverse transcriptase	1	2.15	1.87	**2.01**	3.43E-23
**B8ZZW8**	F-BAR and double SH3 domains protein	1	2.16	1.70	**1.93**	1.31E-20
**C9J8S2**	Retinoic acid receptor responder protein 2	1	1.86	1.69	**1.78**	3.71E-16
**C9JDK1**	Potassium channel subfamily K member 2	1	1.86	1.67	**1.77**	7.32E-16
**Q96PV6**	Leukocyte receptor cluster member 8	1	1.81	1.68	**1.75**	2.39E-15
**Q8NCR1**	VPS53 protein	1	1.78	1.66	**1.72**	1.62E-14
**Q8N122**	Regulatory-associated protein of mTOR	1	1.74	1.67	**1.71**	2.86E-14

## Data Availability

The data used to support the findings of this study are included within the article.

## Supplementary Materials

Supplementary Materials Sup. VPS53 protein level was not altered after GZFLW treatment in normal endometrial cell. Western analysis of VPS53 in normal endometrial in the presence of GZFLW or not. Actin as an internal control.
