# Supplementary material for: GZFLW Induces Apoptosis of Ectopic Endometrial Stromal Cells via Promoting VPS53 Protein Stability
Source: Evid Based Complement Alternat Med. 2018 Dec 13;2018:1293630. doi: 10.1155/2018/1293630 (PMC6311267; doi:10.1155/2018/1293630)
Supplement: Supplementary Materials — Sup. VPS53 protein level was not altered after GZFLW treatment in normal endometrial cell. Western analysis of VPS53 in normal endometrial in the presence of GZFLW or not. Actin as an internal control. [file 1293630.f1.pdf]

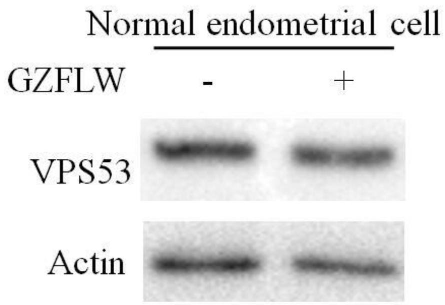

Sup. VPS53 protein level was not altered after GZFLW treatment in normal endometrial cell. Western analysis of VPS53 in normal endometrial in the presence of GZFLW or not. Actin as an internal control.
